# Social Inequalities and Geographical Distribution in Caries Treatment Needs among Schoolchildren Living in Buenos Aires City: A Cross-Sectional Study

**DOI:** 10.3390/dj12100325

**Published:** 2024-10-09

**Authors:** Fiorella Ventura, Maria Rocio Lazzati, Pablo Andres Salgado, Glenda Natalia Rossi, Thomas G. Wolf, Aldo Squassi, Guglielmo Campus

**Affiliations:** 1 Universidad de Buenos Aires, Facultad de Odontología, Cátedra de Odontología Preventiva y Comunitaria—Instituto de Investigaciones en Salud Pública, (Department of Preventive and Community Dentistry, Faculty of Dentistry, University of Buenos Aires), Marcelo Torcuato de Alvear 2142, Buenos Aires C1122AAH, Argentina; fiorella.ventura@odontologia.uba.ar (F.V.); pablo.salgado@odontologia.uba.ar (P.A.S.); glerossi@yahoo.com.ar (G.N.R.); aldo.squassi@odontologia.uba.ar (A.S.); 2 Universidad de Buenos Aires, Facultad de Odontología, Instituto de Investigación en Salud Pública (Institute of Public Health Research, Faculty of Dentistry, University of Buenos Aires), Marcelo Torcuato de Alvear 2142, Buenos Aires C1122AAH, Argentina; rocio.lazzati@odontologia.uba.ar; 3 Universidad de Buenos Aires, Facultad de Odontología, Departamento de Microbiología y Parasitología, (Department of Microbiology and Parasitology, Faculty of Dentistry, University of Buenos Aires), Marcelo Torcuato de Alvear 2142, Buenos Aires C1122AAH, Argentina; 4 Department of Restorative, Preventive and Pediatric Dentistry, School of Dental Medicine, University of Bern, Freiburgstrasse 7, 3010 Bern, Switzerland; thomas.wolf@unibe.ch; 5 Department of Operative Dentistry, Johannes Gutenberg University Medical Center, University of Mainz, Saarstraße 21, 55122 Mainz, Germany; 6 Consejo Nacional de Investigaciones Científicas y Técnicas Godoy Cruz 2290, Buenos Aires C1425FQB, Argentina; 7 Department of Cariology, Saveetha Dental College and Hospitals, SIMATS, 162 Poonamallee High Rd, Velappanchavadi, Chennai 600077, Tamil Nadu, India

**Keywords:** caries epidemiology, caries treatment needs index, social inequalities, children, South America, Argentina, paediatric dentistry, public health dentistry

## Abstract

Background: Caries experience in Argentine children is quite high and no national data are present. Aim: This cross-sectional observational survey aimed to assess the oral health status of schoolchildren in Buenos Aires (CABA) using CTNI and to evaluate the ecological association between health inequalities and caries prevalence. Design: Data were collected, via a clustered procedure, during the 2016–2018 school years. Children were examined at school to determine dental status according to the CTNI performed under standardized conditions, using light, dental mirrors, WHO probes and magnification (2.5×). Secondary sources were used to acquire socio-demographic and epidemiological data. A hierarchical cluster analysis was carried out with the municipalities of the CABA as the dependent variable and the socio-demographic factors. Results: The study population consisted of 62,904 schoolchildren, 43,571 of whom were enrolled into the study. Total caries prevalence was 67.78% while severe caries prevalence was 28.29%, which was statistically associated with the year of the survey (*p* < 0.01). Four territorial conglomerates from a socio-epidemiological standpoint outline homogeneous areas of social risk; cluster 1 had the highest social risk. The prevalence of severe caries (CTNI 7–14) was highest in cluster 1 (44.7–53.2% of the population). Conclusion: The social inequalities between municipalities of the CABA were statistically associated with high inequalities in oral health needs.

## 1. Introduction

Caries distribution presents similar features to many other social gradients in health and its distribution is not uniform in different populations as it is linked to several macro and micro socio-economic variables such as gross national income (GNI) of the country, the employment rate, and immigrant status [1]. Oral conditions, particularly dental caries and their consequences, have major public health impacts for the entire global population and lifestyle changes are essential to ensure better health [2].

The social determinants of health (SDOH) refer to all the environmental factors that have an impact on well-being. Micro (individual) and macro (community) social determinants have been identified as strong risk factors for poor oral health [3]. The implementation of strategies that target modifiable factors at the micro level proved inadequate for enhancing oral health and reducing inequities unless accompanied by upstream policies that address macro-level factors [4]. Understanding these determinants and their mechanisms of correlation might support the application of efficient and effective upstream oral health policies and prevention strategies.

Social inequalities experienced or exposed to during the stages of child development negatively impact adults, contributing to the perpetuation of social inequalities in adult life [2,5].

Children whose families were in the lowest household income quintile, regardless of immigrant status, had the highest caries experience. These children often report a low frequency of daily toothbrushing, high consumption of sugar-containing foods and infrequent dentist check-ups [3,4,5,6,7,8].

Argentina is the second-largest country in South America after Brazil, with a population of 46,234,830, an increase of more than 15% from the previous census [9]. The country is highly urbanized with the capital metropolitan area “Ciudad Autónoma de Buenos Aires (CABA)” containing around 14 million inhabitants. The country has the highest inflation rates in the world at 221.4% in 2023. Approximately 43% of Argentina’s population lives below the poverty line as reported in 2023 [10]. Caries figures in children are quite high in the country, and no national data are present. Local data such as in Mendoza show a caries prevalence of almost fifty percent [11,12,13,14]. The indicators available for the conceptual and operational management of dental caries must be coherent and must enable the process of clinical development of the disease, from its early stages to its complications, to be accurately identified [11,12,13,14]. The Caries Treatment Needs Index (CTNI) was developed and applied in 1993 and is based on the interaction of two axes: the progression of the lesion and the technological resources required to control the risk of caries [13]. The severity component identifies the process of tissue deterioration of the caries lesion, from a clinical threshold that recognizes initial caries in the enamel to subsequent progression to cavitation. The score is expressed as the number of oral quadrants with visible lesions. The technological axis includes the risk component and the available technological development component. Technological development is based on contextualized scientific evidence and is expressed in terms of appropriate strategies and their application per mouth unit and tooth, according to the magnitude recorded in the dental quadrants. Since its inception, the CTNI has been evaluated in comparison with other caries indicators and has shown a higher correlation than other indicators such as the ICDAS, confirming the applicability of the CTNI to different populations and age groups [11,12,13,14,15,16].

Based on this premise, the present study was designed as a cross-sectional observational survey on the oral health of schoolchildren living in the CABA area using the CTNI as a caries indicator. Secondary aims of the study were to describe and assess the ecological association of health inequalities and caries data evaluating the potential spatial correlation between these data.

## 2. Materials and Methods

### 2.1. Design of the Study and Sample Size

The study was designed as an analytic cross-sectional study including clinical assessment and collection of socio-economic variables. The study proposal was approved in 2013 and again in 2019 by the Ethical Committee “Ethical Committee—School of Dentistry of the University of Buenos Aires” (Comité de Ética FOUBA 29/05/2013-52 and N° 019/2019-CETICA-FOUBA 09/10/2019). The area selected for the study was the Autonomous City of Buenos Aires (CABA). Two distinct observational units were selected: the city communes, representing the city’s political and administrative management units, and three cohorts of first-grade schoolchildren enrolled in public schools. The study was developed within the framework of a technical cooperation agreement between the Ministries of Education and Health, the government of the city of Buenos Aires and the Faculty of Dentistry of the University of Buenos Aires. Data were collected from primary (clinical examination) and secondary sources (socio-economic backgrounds). The data were collected during the 2016–2018 schoolyears. In the CABA area, a total of 62,904 6-year-old children attended 1st-grade primary schools (n = 1307) [17,18]. A clustered sample procedure was followed; the schools were computed into a list and then selected with a regular interval of two schools. An expansion factor was applied to weight the sample according to the proportion of schoolchildren per commune [19]. Parents and/or caregivers of the children received a leaflet explaining the aim of the study and requesting via signed consent the participation of each schoolchild in the study. Participation in the study was entirely voluntary.

### 2.2. Clinical Assessment and Calibration Procedures

Children were examined at school to determine dental status according to CTNI criteria (Table 1), performed under standardized conditions, using light, dental mirrors, WHO probes and magnification (2.5×). The dental exams were performed by 25 calibrated researchers. The calibration was carried out prior to the start of the trial with the reference examiner.

The calibration strategy consisted of the following:

(a) Expository class (2 h) with photographs (n = 36) aimed at the recognition of the categories established in the CTNI and the cut-off points between the different categories and the protocol to carry out the diagnosis.
(b) Caries detection using extracted teeth (n = 30) (ex vivo 2 h). The specimens were examined after drying the surfaces with compressed air and under adequate lighting. Each operator recorded the observed findings according to the lesion and activity criteria for each tooth surface. The results were then discussed with the benchmark examiner.
(c) Clinical practice (20 h), which included the following steps:
  - Assignment to each examiner of 6 volunteer children who provided a balanced number of dental surfaces with CTNI codes.
  - Observation and recording of the findings in an ad hoc spreadsheet. The visual–tactile clinical examination was performed with a frontal light, WHO probes, magnification (2.5×) and air drying of the surfaces.
  - Re-evaluation (one week later) of each patient by the reference examiner and recording of findings.

Inter- and intra-examiner reliability was assessed by comparing the benchmark and examiner results and the percentage of agreement using Cohen’s kappa statistic for healthy surfaces, enamel and dentinal lesions.

Secondary sources were used to acquire socio-demographic and epidemiological data [17,18]. The 2016–2018 databases were searched and the following data were retrieved: employment percentage of the head of the household; percentage of the households with precarious tenure; percentage of children having access to only the public health system; percentage of children attending public schools; % of households with a head that has completed primary school; % unemployed; per capita family income (z score); and overcrowding. The infant mortality rate was retrieved from the Vital Statistics of the General Directorate of Statistics and Censuses (Ministry of Finance GCBA).

### 2.3. Data Analysis

All data were entered into a Microsoft Excel^®^ version 16.78.3 spreadsheet. On the basis of the clinical examination, besides the ICDAS score, the CTNI [13] was also computed (Table 1). The CTNI is based on the interaction of two axes: one based on the progression of the lesion and the other on the technological resources needed to control the risk of dental caries. The progression axis identifies the magnitude of severity and extent. The magnitude of severity component identifies the process of tissue involvement of the dental caries lesion, moving from the white spot lesion to the subsequent progression of the lesion towards cavitation. The number of oral quadrants with visible lesions represents the extent of the magnitude in the “oral unit”. The risk component and the available technological development component are included in the technology axis. The risk component is the result of the identified variables, while the technological development is based on contextualized scientific evidence and is expressed as the appropriate strategies and their application per oral unit and tooth, according to the extent recorded in the dental quadrants [13].

No instrumentation is required for its determination. It is a visual index whose only requirements are good lighting and a clean mouth. Total caries prevalence and severe caries prevalence were calculated as the absolute number and percentage of children with a CTNI score greater than 2 (caries prevalence) and the absolute number and percentage of children with a CTNI score greater than 7 (severe caries prevalence).

The Ciudad Autónoma de Buenos Aires (CABA) is divided into 15 municipalities named communes. A hierarchical cluster analysis was carried out with the municipalities of the CABA as the dependent variable and the socio-demographic factors as mentioned above. Hierarchical cluster analysis is a clustering technique used to identify patterns and similarities in a data set. In this case, nearest neighbor clustering was used, where each observation is first assigned to its cluster and then clusters are merged based on their proximity. The distances between observations were calculated using the Euclidean distance measure and the values were standardized using z-scores [20] to ensure that the variables have the same scale and contribute equitably to the calculation of distances. The results were visualized using a dendrogram. The percentage distribution of students requiring caries treatment in each cluster was then calculated and analyzed by z-scores as a comparison in proportion [20].

## 3. Results

The study population consisted of 62,904 schoolchildren attending first-year primary schools in the 15 municipalities of the CABA during the years 2016 and 2017. Overall, 17,343 children were examined in 2016, 12,425 in 2017 and 13,803 in 2018 (69.2% of the total study population of first-graders) (Table 2).

Inter-examiner reliability, measured by Cohen’s Kappa, ranged from 0.73 (_95%_CI 0.68–0.78) to 0.86 (_95%_CI 0.79–0.91) for sound and from 0.79 (_95%_CI 0.74–0.83) to 0.83 (_95%_CI 0.76–0.90) for extensive distinct cavities with visible dentine. Intra-examiner reliability ranged from 0.83 (_95%_CI 0.79–0.88) to 0.90 (_95%_CI 0.84–0.95) for sound teeth and from 0.83 (_95%_CI 0.80–0.86) to 0.89 (_95%_CI 0.85–0.93) for distinct cavities with visible dentine.

The sex distribution of children across three years was similar (Pearson χ^2^_(2)_ = 0.87 *p* = 0.65); females comprised 48.19% in 2016, 47.65% in 2017 and 47.86% in 2018. Total caries prevalence was 67.78% while severe caries prevalence was 28.29%. No statistically significant association was discernible between sex and caries prevalence and the prevalence of severe caries. Severe caries prevalence was statistically associated with the year of the survey (Pearson χ^2^_(2)_ = 17.77 *p* < 0.01); in 2016, the prevalence of severe caries (CTNI score 8–14) was 38.27% compared to 28.96% in 2017. The health coverage was statistically significantly associated with total and severe caries prevalence (*p* < 0.01) (Table 2). The socio-epidemiological characteristics of the subjects across the different municipalities are displayed in Table 3.

The infant mortality rate ranged from 3.9 in municipality 2 to 8.8 in municipality 4. The overall Z score fluctuates from −1.78 (municipality 8) to 1.82 (municipality 14). Starting from these data, four hierarchical clusters were identified according to socio-epidemiological variables (Table 4).

**Figure 1:**
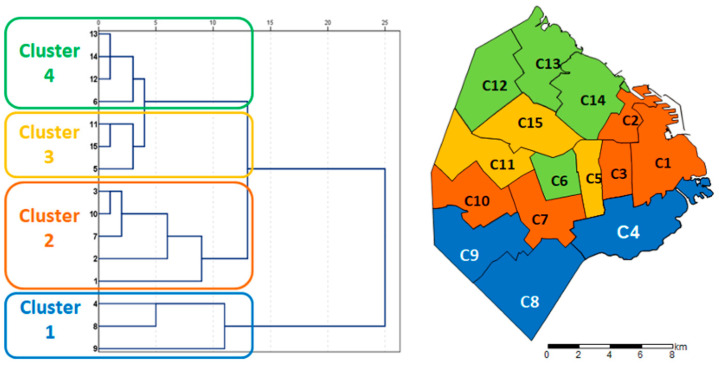
Geographical areas of the city of Buenos Aires (Argentina) and distribution according to four different clusters.

The data delineate four territorial conglomerates from a socio-epidemiological standpoint, outlining homogeneous areas of social risk, and the map of the four different clusters in the Buenos Aires Area is displayed in Figure 1.

**Figure 2:**
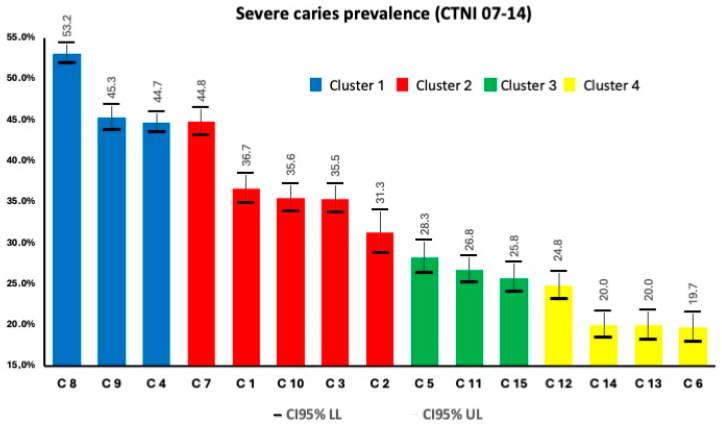
Severe caries prevalence (CTNI 7–14) across the four different clusters.

Cluster 1 exhibits maximum and minimum values surpassing the city’s mean across various variables. These include households with precarious tenure, exclusive reliance on the public health system, households headed by individuals with only primary school education, overcrowding, unemployment and per capita household income. The region encompassing municipalities 6/12/13/14 (cluster 4) is characterized by the most favorable indicators in the Buenos Aires area (Figure 2).

The prevalence of severe caries (CTNI 7–14) was highest in cluster 1 (44.7–53.2% of the population) with a percentage of schoolchildren in need of treatment of almost 80%; at the other extreme, cluster 4 presents 55% of schoolchildren in need of caries treatment. The other indicator obtained from primary sources was the % of public health system-only access, which shows a distribution compatible with the rest of the indicators, with 71.1% of schoolchildren in cluster 1 having exclusive public health coverage, 55.7% in cluster 2, 38.5% in cluster 3 and 31.6% in cluster 4. The defined geographical areas showed a heterogeneous distribution of the need for severe caries treatment, this being more frequent in the most socially disadvantaged areas.

## 4. Discussion

Oral diseases are among the leading non-communicable diseases globally, with dental caries, periodontal disease, edentulism, and oral cancer being the most prevalent [21]. Children, especially those living in low- and middle-income countries, have the highest prevalence of oral diseases worldwide. The global prevalence of dental caries in primary and permanent dentition in children is 46.2% and 53.8%, respectively [22]. The results of this study reveal that the prevalence of caries in the city of Buenos Aires is higher than the global average.

Students are grouped into different educational circuits based on their socio-economic level. The concept of an educational circuit refers to the classification of educational institutions based on similarities in their characteristics, such as the socio-economic level and socio-cultural capital of their students, the profile of the teachers, the infrastructure, the pedagogical models, and the organization, among other aspects. Those belonging to less privileged socio-economic strata tend to be in circuits of lower quality. In our study, an unequal distribution in the prevalence of dental caries lesions was observed in the different regions into which the city of Buenos Aires was divided, with an annual increase that is more pronounced in the districts with less favorable socio-economic indicators.

The findings of the analysis on socio-economic variables and their relationship with oral health revealed a significantly higher level of dental disease in groups with social vulnerability, with a greater number of children at high risk and with cariogenic activity in neighborhoods characterized by higher social risk [23,24,25]. A similar figure was observed in the present survey of almost all children attending the first grade of public schools, which highlights the inequalities between sectors with differing socio-economic indicators.

In Argentina, as well as in several developing countries, a clear distinction is made between state-managed and private schools. These two school networks have notable differences derived from their historical origins. The choice to focus this study on state-managed schools implies an intention to examine the lower-income strata concentrated in these educational institutions. The type of health coverage plays a decisive role in access to healthcare services. Social security represents additional coverage provided by social security institutions and, in this context, acts as a protection mechanism for workers employed in the formal market. Being a beneficiary of social security becomes an indirect indicator of formal employment. The marked prevalence of informal employment contributes to exacerbating existing labor instability, lack of institutionalization, and limited access to technology and markets. This set of factors allows us to link the prevalence of caries in individuals without medical coverage not only to the lack of attention to this pathology due to difficulties in accessing health services but also to the social vulnerability that this situation represents. It is necessary to continue designing and implementing epidemiological studies, with standardized methodologies, covering different geographical areas of the Argentine Republic, to have information that can assist policymakers in formulating health strategies that can modify disease profiles.

The outcomes of this survey evidenced heterogeneities in the distribution of dental care needs, which were higher in the conglomerates of municipalities with the most unfavorable socio-epidemiological indicators.

Cluster number one shows values above the overall values of the Buenos Aires area for households with precarious tenure, access to the public health system exclusively, households with a head that has completed primary school, overcrowding, unemployment and per capita family income, showing values of greater vulnerability.

The relevance of this study lies in the fact that identifying the different variables related to the health situation in a defined geographic population space allows the identification of gaps in which intervention can be carried out in a specific and differentiated way, as well as monitoring the changes that are going to take place and improving the problems identified in each territory, according to its particular characteristics. The data collected in this work at the local level could constitute valuable input for management.

Practical and economical approaches that have been proposed in a study within a developing Caribbean nation—providing direct access to dental therapists, offering dedicated emergency and routine care appointments, delivering oral health campaigns, partaking in community health fairs, social media engagement, forming collaborative partnerships with other dental and healthcare professionals and closely following the Pan American Health Organization’s recommendations on oral health management [26]—could be very useful interventions within the Argentinian context.

## 5. Conclusions

The state-run schools located in the municipalities with the most unfavorable socio-epidemiological characteristics concentrate a higher proportion of first-grade schoolchildren in need of caries treatment.

The inequalities identified in oral health needs show the social inequalities between municipalities of the CABA.

## Figures and Tables

**Table 1:** Caries Treatment Needs Index (CTNI).

CODE	Clinical Situation	Unit of Analysis	Treatment Plan
00	*Sound teeth with history of preventive measures*	Mouth	Preventive program: low or moderate caries risk
01	*Sound teeth without history of preventive measures*		Preventive program: low or moderate caries risk
02	*Presence of initial caries lesions*		Preventive program: high caries risk
03	*Presence of cavitated lesions affecting enamel and/or dentine*	1 quadrant	Preventive program: high caries risk + restorative treatment
04		2 quadrants	Preventive program: high caries risk + restorative treatment
05		3 quadrants	Preventive program: high caries risk + restorative treatment
06		4 quadrants	Preventive program: high caries risk + restorative treatment
07	*Presence of cavitated lesions affecting enamel and/or dentine with pulp involvement*	1 quadrant	Preventive program: high caries risk + restorative treatment + pulp treatment
08		2 quadrants	Preventive program: high caries risk + restorative treatment + pulp treatment
09		3 quadrants	Preventive program: high caries risk + restorative treatment + pulp treatment
10		4 quadrants	Preventive program: high caries risk + restorative treatment + pulp treatment
11	*Presence of extensive cavitated lesions without possibilities of restorative treatment or presence of abscess or fistula*	1 quadrant	Preventive program: high caries risk + restorative treatment + surgical treatment and eventual rehabilitation
12		2 quadrants	Preventive program: high caries risk + restorative treatment + surgical treatment and eventual rehabilitation
13		3 quadrants	Preventive program: high caries risk + restorative treatment + surgical treatment and eventual rehabilitation
14		4 quadrants	Preventive program: high caries risk + restorative treatment + surgical treatment and eventual rehabilitation

**Table 2:** Distribution of the sample regarding caries prevalence (CTNI score > 2) and severe caries prevalence (CTNI > 7) across sex, year of recruitment and living area. The association between caries data and demographic variables was assessed using the Pearson chi-square test.

		Caries Prevalence		Severe Caries Prevalence	
		CTNI Score (0–2) *n* (%)	CTNI Score (3–14) *n* (%)	CTNI Score (0–7) *n* (%)	CTNI Score (8–14) *n* (%)
Sex	Males	7366 (52.46)	15,321 (51.88)	16,211 (51.88)	6476 (52.54)
	Females	6674 (47.54)	14,210 (48.12)	15,035 (48.12)	5849 (47.46)
		*Pearson χ^2^_(2)_ = 1.30 p = 0.26*		*Pearson χ^2^_(2)_ = 1.55 p = 0.21*	
Year	2016	5655 (40.28)	11,688 (39.58)	12,626 (40.41)	4717 (38.27)
	2017	4008 (28.55)	8417 (28.50)	8856 (28.34)	3569 (28.96)
	2018	4377 (31.18)	9426 (31.92)	9764 (31.25)	4039 (32.77)
		*Pearson χ^2^_(2)_ = 2.83 p = 0.24*		*Pearson χ^2^_(2)_ = 17.77 p < 0.01*	
Living Area	CABA 1	4125 (29.38)	13,487 (45.67)	11,106 (35.54)	6506 (52.79)
	CABA 2	7830 (55.77)	13.467 (45.60)	16,239 (51.97)	5058 (41.04)
	CABA 3	2085 (14.85)	2577 (8.73)	3901 (12.48)	761 (6.17)
		*Pearson χ^2^_(2)_ = 1.2 × 10^3^ p < 0.01*		*Pearson χ^2^_(2)_ = 1.2 × 10^3^ p < 0.01*	
Health	Public	4775 (34.07)	17,819 (60.47)	13,937 (44.70)	8657 (70.35)
	Private/social security	9150 (65.28)	11,386 (38.64)	17,011 (54.56)	3525 (28.65)
	No replies	92 (0.66)	263 (0.89)	232 (0.74)	123 (1.00)
		*Pearson χ^2^_(2)_ = 2.7 × 10^3^ p < 0.01*		*Pearson χ^2^_(2)_ = 2.4 × 10^3^ p < 0.01*	

**Table 3:** Socio-epidemiological characteristics variables of the different municipalities. The variables are expressed as percentages.

Municipality	Infant Mortality Rate	% Employment of the Head of the Household	% Households with Precarious Tenure	% Public System Only	% Attend Public Education	% Completed Primary School	% Unemployed	Z Score (IPCF)	% Overcrowding
1	7.0	74.3	16.3	30.2	70.1	14.6	8.7	0.04	20.6
2	3.9	67.4	12.0	6.8	46.8	5.5	5.6	1.43	4.7
3	6.7	72.3	9.4	19.3	58.3	12.9	7.5	−0.21	13.6
4	8.8	65.4	23.0	36.8	75.0	24.3	12.7	−1.14	19.7
5	6.6	69.9	9.1	11.6	56.6	8.2	6.2	0.31	7.6
6	5.0	72.3	9.0	7.7	50.8	6.0	6.0	0.64	3.2
7	6.8	71.2	12.9	24.7	61.0	13.2	8.6	−0.57	12.3
8	8.7	67.1	26.3	46.4	74.2	29.1	13.7	−1.78	22.9
9	8.4	67.1	12.2	25.4	57.9	19.6	8.0	−1.07	11.2
10	4.9	69.0	8.9	17.9	55.0	11.9	6.9	−0.62	6.4
11	6.3	69.8	7.8	13.5	52.4	10.3	8.2	−0.22	7.2
12	6.1	70.8	9.7	10.6	51.9	10.3	7.2	0.17	5.8
13	4.8	71.0	9.6	6.1	41.1	4.7	4.9	1.21	3.6
14	4.1	72.5	10.4	5.6	38.0	5.0	5.0	1.82	3.7
15	6.9	6.91	71.2	12.9	59.8	11.4	7.4	0.00	6.7

*Source: Annual Household Survey from 2016, 2017, 2018.* *Direcciòn General de Estadìstica y Censos.*

**Table 4:** Employment rate, rate of households with precarious tenure, rate of public health system-only access, rate of public education, rate of primary school completion, rate of unemployment and rate of overcrowding of the four different clusters consisting of geographical areas of the city of Buenos Aires.

Clusters *	% Employment of the Head of the Household	% Households with Precarious Tenure	% Public Education (3 years or more)	% Public Health System Only	% Completed Primary School	% Unemployed	Z Score (IPCF)	% Overcrowding
Cluster 1	65.8	20.5	69.0	36.2	24.3	11.4	−1.330	17.9
Cluster 2	70.3	10.3	56.3	12.6	10.0	7.3	0.030	7.1
Cluster 3	70.8	11.9	58.3	19.8	11.6	7.5	0.013	11.5
Cluster 4	71.7	9.7	45.5	7.5	6.5	5.8	0.958	4.1
Buenos Aires	69.9	12.7	56.6	18.4	12.5	7.8	0.00	9.9

* Cluster 1 (municipalities 4, 8, 9); cluster 2 (municipalities 1, 2, 3, 7, 10); cluster 3 (municipalities 5, 11, 15); cluster 4 (municipalities 6, 12, 13, 14).

## Data Availability

The raw data are available on request to the corresponding author.
